# Self-Report Stress Measures to Assess Stress in Adults With Mild Intellectual Disabilities—A Scoping Review

**DOI:** 10.3389/fpsyg.2021.742566

**Published:** 2021-10-25

**Authors:** Martina de Witte, Roel Kooijmans, Maria Hermanns, Susan van Hooren, Kim Biesmans, Maaike Hermsen, Geert Jan Stams, Xavier Moonen

**Affiliations:** ^1^Research Institute of Child Development and Education, University of Amsterdam, Amsterdam, Netherlands; ^2^Faculty of Health and Vitality, HAN University of Applied Sciences, Nijmegen, Netherlands; ^3^KenVaK, Research Centre for the Arts Therapies, Heerlen, Netherlands; ^4^Stevig, Expert Centre for People With Mild Intellectual Disabilities, Gennep, Netherlands; ^5^Koraal Center of Expertise, Sittard, Netherlands; ^6^Faculty of Healthcare, Zuyd University of Applied Sciences, Heerlen, Netherlands; ^7^Faculty of Psychology and Educational Sciences, Open University, Heerlen, Netherlands

**Keywords:** stress, state anxiety, self-report measures, stress assessment, mild intellectual disabilities, borderline intellectual functioning, scoping review, stress scale

## Abstract

Stress has a major negative impact on the development of psychopathology and contributes to the onset of adverse physical conditions. Timely recognition and monitoring of stress-related problems are therefore important, especially in client populations that are more vulnerable to stress, such as people with mild intellectual disabilities (MID). Recent research on the use of physiological measures to assess stress levels emphasize that, in addition to these measures, self-report instruments are necessary to gain insight into the individual perception and impact of stress on daily life. However, there is no current overview of self-report stress measures that focus on the experience of stress in the present moment or in daily life. To provide an overview of the existing self-report stress measures for clinicians and researchers, a scoping review was conducted. In addition, to advise clinical professionals on the use of self-report measures of stress for people with MID, the results of an expert consultation were used to refine the preliminary findings. A systematic scoping literature search resulted in a total of 13 self-reported stress measures that met the final inclusion criteria, of which three were developed specifically for assessing stress in adults with MID (GAS-ID, LI, and SAS-ID). For each included self-report stress measure, the psychometric quality, assessment procedure, and suitability for adults with MID were reported. These were supplemented by the findings from the expert consultation. Implications for clinical practice on the use of self-report stress measures, particularly for people with MID, are discussed. Recommendations for future research and development are given.

## Introduction

Recognizing a person's stress-related problems is increasingly important, as ever more evidence on the adverse effects of stress on health and well-being is accumulated. High stress levels are regarded as an important risk factor for the onset and progression of a wide range of physical and emotional problems, such as cardiovascular diseases, cancer, anxiety disorders, depression, and burnout (Steptoe and Kivimäki, 2012; Australian Psychological Society, 2015; American Psychological Association, 2017). Nevertheless, the literature reports that it is difficult for many people to both understand the destructive impact of daily life stress experiences (Casey, 2017; de Witte et al., 2020a) and to reduce or cope with stress without any professional support (World Health Organization, 2010). This is especially the case for adults with mild intellectual disabilities (MID), as they experience stress more frequently in daily life than people without intellectual disabilities (Emerson, 2003; Hatton and Emerson, 2004; Schuengel and Janssen, 2006; World Health Organization, 2010). In addition, people with MID have also been found to have fewer resources to cope with daily life stress experiences (Lunsky and Benson, 2001; Hartley et al., 2009a; Scott and Havercamp, 2014).

## Measuring the Concept of Stress

When we use the term “stress” in the present study, we are referring to a negative stress experience as a response to a stressful condition, event, or situation (a stressor). Stress is defined by Aldwin (2007) as the quality of an experience produced by a person-environment transaction that, through either overarousal or underarousal, results in psychological or physiological distress (Aldwin, 2007; Riley and Park, 2015). Responses to stress are related to physiological arousal and emotional states, and the underlying systems of these responses regulate and affect each other in times of stress (McEwen and Gianaros, 2010; Linnemann et al., 2017; de Witte et al., 2020a). The physiological response to stress implies the activation of the hypothalamic-pituitary adrenal (HPA) axis and, due to the release of adrenalin and noradrenalin, increased activity of the sympathetic nervous system. This in turn results in increased physiological arousal, such as heart rate (HR), blood pressure, and cardiac output (Bally et al., 2003; Pfaff et al., 2007). Stress-related emotional states can be defined in terms of subjective worry, nervousness, and restlessness (Cohen et al., 1983; Pritchard, 2009; Akin and Iskender, 2011; Pittman and Kridli, 2011), and have many similarities with “state anxiety” as an outcome. Accordingly, many researchers describe state anxiety as an emotional response to an individual's perception of a stressful experience (e.g., Hook et al., 2008; Koelsch et al., 2011). In this review, we therefore regard state anxiety as a stress-related outcome. Stress-related outcomes can be measured by means of biomarkers related to physiological arousal (physiological measures) and by assessing people's emotional states related to stress experiences (psychological measures). Empirical studies on stress use either physiological or psychological measurement methods (proxy or self-reports) or a combination of both (Kim et al., 2018) for the measurement of stress-related outcomes.

Although there is a large body of knowledge concerning the immediate effects of stress on physiological arousal, as indicated by several biomarkers like HR, blood pressure, heart rate variability (HRV), and hormone levels (Chandola et al., 2010; Föhr et al., 2017; Kim et al., 2018), increased physiological arousal does not automatically translate to elevated levels of perceived stress. It can also signal, for example, that a person is positively excited or deeply focused (Csikszentmihalyi, 2000; Pfaff et al., 2007; Rheinberg and Engeser, 2018). When examining subjective stress levels, many researchers therefore emphasize the importance of assessing the subjects' perceived emotional state in relation to stress, to help interpret physiological markers of arousal.

Both proxy-reported and self-reported information are used to examine psychological stress-related outcomes, such as people's emotional states (Crawford et al., 2006). Proxy reports refer to information about an individual given by significant others, such as relatives or caretakers. These are often used as an alternative when obtaining self-reported information is not a viable option, for instance when the respondent is not able to communicate verbally (Moore, 1988; Miller and Tucker, 1993; Emerson et al., 2013). Evidence suggests that proxy reports may be less accurate and less sensitive, compared to self-reported information (Moss et al., 1996; Scott and Havercamp, 2018).

### Perceived Stress in Adults With Mild Intellectual Disabilities

MID is a neurodevelopmental disability characterized by deficits in intellectual and adaptive functioning skills (American Psychiatric Association, 2013). The term MID generally refers to people with limited intellectual capacities and adaptive skills with IQ scores in the range from 55 to 70, and may in some definitions include persons with “borderline intellectual functioning” (IQ 70–85; Kaal et al., 2015; Wieland and Zitman, 2016).

Adults with MID experience more stress in daily life than people without intellectual disabilities (Bramston and Mioche, 2001; Emerson, 2003; Hatton and Emerson, 2004; Schuengel and Janssen, 2006; World Health Organization, 2010; Casey, 2017; de Witte et al., 2020b). Daily life stressors that impact many lives of people with MID include difficulties with social interactions, experienced lack of control over minor daily and major life decisions, and occupational stress (Hartley et al., 2009b; Dulin et al., 2013; Scott and Havercamp, 2014). Next to common daily life stressors, people with MID have a higher risk of experiencing adversity in childhood, such as abuse and neglect. Mason-Roberts et al. (2018) found that 42.4% of their study participants with mild and moderate ID reported multiple traumatisation in both childhood and adulthood. In studies by Santoro et al. (2018) and Vervoort-Schel et al. (2021), over 80% of children and adults with intellectual and developmental disabilities experienced one or more adverse childhood experiences (ACE). Childhood and later life adversity have been shown to cause chronic overactivity of stress systems (e.g., Van Der Kolk, 2014).

The extent to which daily life stressors and chronic stress lead to perceived psychological stress is moderated in large part by the coping skills and resilience factors of the person facing the stressor. Psychological stress is defined by Cohen et al. (1983) as the degree to which individuals perceive that demands exceed their ability to cope. People with MID have been shown to have greater difficulty coping with stress in daily life and the effects of adversity than adults without intellectual disabilities (American Psychiatric Association, 2013). Adults with MID often seem to lack social support and self-efficacy, important factors for coping with stress (Abbaszadeh and Sardoie, 2016; Seyed et al., 2017; Everly and Lating, 2019). As in the general population, stress experienced by adults with MID is linked to many negative mental health outcomes (Hulbert-Williams and Hastings, 2008; Hartley et al., 2009a,b; Scott and Havercamp, 2014). Persistent or chronic stress in adults with MID can lead to maladaptive coping strategies and detrimental mental and physical health conditions such as depression (Hartley et al., 2009a,b), impaired cognitive functions (Heyman and Hauser-Cram, 2015), physical health problems (Lunsky, 2008), and substance abuse (Didden et al., 2009).

### Psychological Stress Measures for Adults With MID

Both proxies and persons with MID themselves can provide information on the occurrence of stressful situations for the person with MID. There are some concerns with gaining reliable and accurate information from people with MID through self-reports, that mainly originate from the nature of their disability. However, as the perception and psychological impact of stress is a highly subjective internal (and thus not observable) state, the validity of proxy stress measures is perceived to be limited as well (Emerson et al., 2013; Scott and Havercamp, 2018).

Several studies have shown that, when compared to self-reported outcomes, proxies tend to overestimate impairment and underestimate health-related quality of life of people with (M)ID (Andresen et al., 2001; Vlot-van Anrooij et al., 2018). So because of the superior accuracy and sensitivity of self-reported information when it comes to the assessment of subjective internal states, researchers in the field of MID generally prefer self-reporting measures above proxy measures to assess the experience of stress in persons with MID (Lindsay and Skene, 2007; Scott and Havercamp, 2018). Additionally, gaining access to thoughts, attitudes and feelings about stress directly at the source, can lead to an enriched knowledge base from which opinions can be formed and interventions for stress reduction implemented (O'Keeffe et al., 2019).

Several studies have shown that, when compared to self-reported outcomes, proxies tend to overestimate impairment and underestimate health-related quality of life of people with (M)ID (Andresen et al., 2001; Vlot-van Anrooij et al., 2018). So because of the superior accuracy and sensitivity of self-reported information when it comes to the assessment of subjective internal states, researchers in the field of MID generally prefer self-reporting measures above proxy measures to assess the experience of stress in persons with MID (Lindsay and Skene, 2007; Scott and Havercamp, 2018). Additionally, gaining access to thoughts, attitudes and feelings about stress directly at the source, can lead to an enriched knowledge base from which opinions can be formed and interventions for stress reduction implemented (O'Keeffe et al., 2019).

High quality self-report measures on mental states, including stress, for adults with MID are few and far between (Glenn et al., 2003; Sams et al., 2006; Kooijmans et al., 2021a). There are many challenges when collecting self-reported data from people with MID that are associated with the nature of the disability, including problems with reasoning, verbal expression, reading, abstract thinking, and judgment (Schalock et al., 2010; American Psychiatric Association, 2013). To accommodate for these challenges, adaptations have to be made to “standard” instrument language, lay-out, and assessment procedures. On the basis of research into the processes underlying the construction of responses to questionnaires and interview questions, authors have put forward recommendations to tailor questionnaire formats and administration procedures to the specific needs of people with ID (e.g., Finlay and Lyons, 2001; Hartley and MacLean, 2008). A recent review by Kooijmans et al. (2021a) highlights that although many recommendations seem common-sense, most are practice-based and lack scientific validation. Examples of recommendations that are more or less evidence-based include the use of Easy Read guidelines to simplify language, the use of Likert scales with a limited number of response options and the sparing use of open-ended questions. The visualization of questions and responses merits attention, as it is generally recommended to use visual supports in the form of pictograms, drawings or photos, while it remains unclear what types of support actually promote understanding instead of causing cognitive overload or confusion (Sutherland and Isherwood, 2016). Few self-report measures are available that incorporate evidence-based adaptations to better suit individuals with intellectual disabilities (Lindsay and Skene, 2007; Scott and Havercamp, 2018). Additionally, Wieland et al. (2012) have identified a number of self-report measurement instruments developed for use in the general population which are suitable for adults with MID.

### Purpose of the Present Study

As persistent stress can lead to the development of psychopathology and severe physical conditions, it is becoming increasingly important to recognize stress-related symptoms in populations known to be more vulnerable to stress, like people with MID. It is therefore critical to gain more insight into the way stress can be assessed in this population. Although advances in the use of physiological measures to assess people's stress levels have added substantial value to stress research, it is no substitute for the use of self-report measures, since the individual's perception of stress is directly related to individuals' emotional states. As stated before, physiological and emotional stress are not necessarily directly related (e.g., Linnemann et al., 2017; Scott and Havercamp, 2018; de Witte et al., 2020b). In order to provide an overview of the existing self-report stress measures and to provide more information about their suitability for adults with MID, we conducted a scoping review. We searched the peer-reviewed literature to identify self-report stress measures. Subsequently, we searched peer-reviewed literature databases and gray literature sources to collect information on several predefined characteristics of these measures. In order to advise clinical professionals on how to correctly use the identified self-report stress measures, expert consultations were held to refine our preliminary findings. Our findings can be applied to research in which stress-related outcomes are measured in both adults with MID as well as those without intellectual disabilities. Results of this scoping review will provide guidance to clinical practitioners to assess perceived stress in adults with MID.

## Method

In order to provide an overview of existing stress self-report measures and describe their properties, we performed a scoping review. A scoping review follows a systematic approach to map evidence or to bundle scientific findings on a topic to identify concepts, theories, sources, and knowledge gaps (Arksey and O'Malley, 2005; Munn et al., 2018; Tricco et al., 2018). Contrary to systematic reviews, scoping reviews can also accommodate gray literature sources, opinions and non-peer-reviewed policy guidelines (Munn et al., 2018). Considering the diverse nature of information sources, risk-of-bias assessment of included sources may often not be appropriate for a scoping review. A scoping view approach matches our research questions, which aim to provide more insights into the different types of self-report measurements and their characteristics, and how they can be used in adults with MID.

For conducting and reporting the review, the authors have followed the guidelines for scoping reviews from the Preferred Reporting Items for Systematic Reviews and Meta-Analyses, Extension for Scoping Reviews (PRISMA-ScR; Tricco et al., 2018).

### Search and Selection Process

#### Search Terms and Sources

Multiple systematic searches were performed with the help of a university information specialist. Engagement of an information specialist to guide a systematic literature search is associated with significantly higher quality of reported search strategies (Rethlefsen et al., 2015). We conducted a computer-based search of the psychological and medical electronic literature databases, including Medline, Academic Search Complete, CINAHL, Cochrane Library, Web of Science, Wiley Online Library, SpringerLink, PiCarta, Academic Search Premier, ScienceDirect, PsycINFO, and Google Scholar. Appropriate key words were identified through exploring the literature on “stress assessment,” “stress questionnaires,” and “stress measures.”

Many previous studies have examined the relationship between state anxiety outcomes and physiological stress-related outcomes (e.g., Hook et al., 2008; Koelsch et al., 2011; de Witte et al., 2020a,b) and defined state anxiety as a stress-related emotional state (Lazarus, 1966; Meijer, 2001; Yang et al., 2011; de Witte et al., 2020a,b). We have therefore included state anxiety as a stress-related outcome in our current study. In addition, we note that in the literature, the concepts of stress and state anxiety are used interchangeably (Lazarus and Folkman, 1984; Wetsch et al., 2009; Pittman and Kridli, 2011; Ozer et al., 2013; Bradt and Dileo, 2014).

We then combined multiple search terms related to stress or state anxiety with terms referring to psychological testing. Appendix A contains an exemplary search string used for the PsycINFO database. Searches were limited to publication dates from 1980 to April 2020. This time frame is consistent with the consensus within the literature that research concerning psychological measures of stress and / or state anxiety commenced in the 1980's (e.g., Cohen et al., 1983; Spielberger et al., 1983). In addition to the online databases, forward and backward searches were conducted by screening the reference lists of included studies, visiting a university testing library, and consulting research experts for “gray” literature. The initial search resulted in the screening of a total of 3,451 studies and an additional 20 measures from forward and backward searches.

#### Selection of the Self-Report Stress Measures

To identify the self-report stress measures that fit the aims of the present study, we applied several selection criteria in two different selection steps. The first step concerned the screening of the studies found. Titles and abstracts of all the English-language peer-reviewed studies were screened for relevance, which means they had to include the terms “stress” or “state anxiety” related to psychological measures. Psychological measures that did not purely target general stress or state anxiety or stress in daily life were excluded, such as measures specifically assessing work stress, long term stress, parenting stress, or stress within the context of a specific medical diagnosis. At this stage, studies were also included in cases where the abstracts did not explicitly state whether the scale used was specifically a self-report stress measure, or whether the outcome measure concerned stress or state-anxiety in general or in daily life. Studies on self-report stress measures in non-English languages were excluded. This selection step ultimately resulted in 75 self-report measures assessing stress or state anxiety in adults. This reduced the number of studies to 25, which were then full-text screened by at least one author. Appendix B contains the complete overview of the self-report stress measures that resulted from this step one selection.

**Figure 1:**
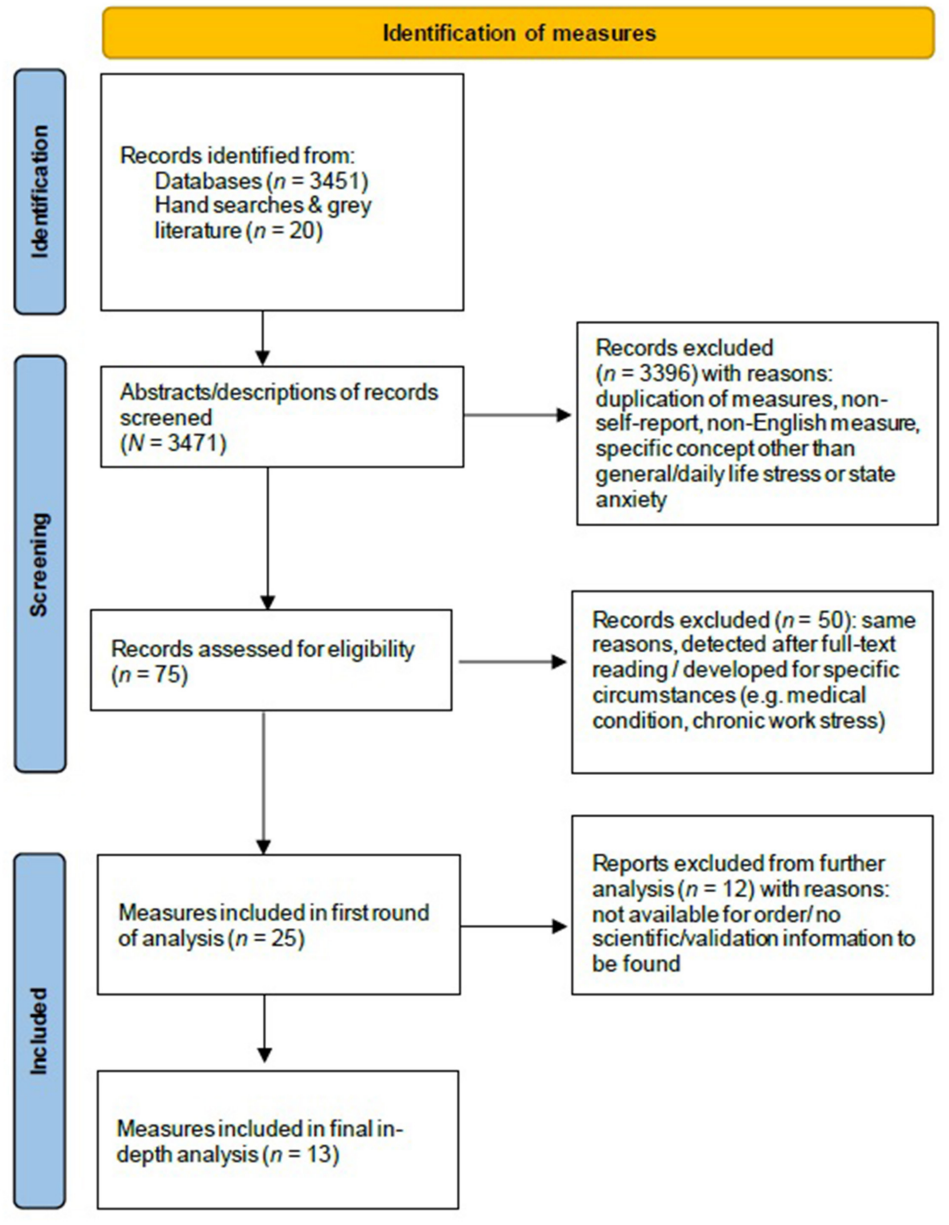
Flow diagram of the selection process.

The second selection step concerned the final inclusion of the self-report stress measures. Therefore, we applied the following criteria: instruments had to (1) be available for order in English, (2) have been applied in (clinical) outcome studies published in peer-reviewed scientific journals, and (3) instructions for assessment of the instrument are available. This selection step was performed by the first three authors (MdW, RK, and MH) independently. Discrepancies were resolved through discussion. This resulted in consensus on the inclusion of 13 self-report measures for further analysis (see Figure 1).

### Evaluation of Included Self-Report Stress Measures

To provide insights into the characteristics and quality of the included self-report stress measures, criteria were formulated to describe their properties. Instrument characteristics relating to the criteria were found in the actual self-report stress measure itself, the user manual, validation studies, and other publications about the self-report measure in peer-reviewed and gray literature. The criteria applied to (1) the psychometric quality of the measure, (2) the assessment procedure of the self-report stress measure, and (3) the suitability for adults with MID. A further definition of the assessment criteria is presented below. Outcomes that relate to each criterion are presented in **Table 2** in the Results section for all instruments.

#### Psychometric Quality

Reliability and validity are considered the main measurement properties of outcome measures used in clinical practice and research (Frost et al., 2007).

##### Reliability

A reliable measure is one that measures a construct consistently across time, individuals, and situations. When defining the psychometric quality of measures, three indicators of reliability are generally considered: test-retest reliability (stability over time), internal consistency (coherence of items with the concepts under study), and interrater reliability (equivalence across different researchers or assessors; Salmond, 2008). Assessing test-retest reliability is typically done by computing Pearson's *r*. A Pearson's *r* of 0.70 or above indicates acceptable alternate-forms reliability (Chiang et al., 2015). For internal consistency, Cronbach's α is most often reported. An α ≥ 0.70 is generally considered adequate, and a value of α ≥ 0.80 is generally considered an indicator of good internal consistency (Allen et al., 2010; Chiang et al., 2015). Interrater reliability concerns the extent to which the different observers are consistent in their judgements. Interrater reliability is often reported as Cronbach's α. For each included self-report measure, we reported the published internal consistency coefficients (Cronbach's α). Manuals were investigated for clear instructions regarding the interpretation of test scores to support objectivity (Moosbrugger and Kelava, 2012).

##### Validity

The term validity refers to the property of an instrument to measure exactly what it proposes. The main criteria and statistical tests for the assessment of validity are used to determine the content, criterion and construct validity of a measure (Frost et al., 2007). Content validity is evaluated to determine whether the instrument items were generated in accordance with relevant theory. To determine the content validity of the self-report measures, it is important that the self-report stress measure contains a clear description of the measuring construct; all terms related to the target group and outcome measure(s) have to be operationalized. We reported whether the self-report stress measure operationalized the key terms appropriately, such as a description of the characteristics of the type of stress measured, and whether the distinction between stress exposition and stress reaction was described (Chiang et al., 2015; Harkness and Monroe, 2016). Moreover, to provide more insights in the validity of the included self-report measures, we also refer to independent validation research and / or assessments by test commissions. Criterion validity refers to the extent to which the measure agrees with an external standard measure. In the case of stress measurement, the outcomes of psychological self-report measures can be, for instance, compared to physiological measures related to stress responses.

Another relevant form of validity concerns construct validity, which refers to the extent to which scores on a measure correlate with the results of a different test. Concurrent validity is a form of construct validity that determines if the measure correlates highly with an established or widely used test already considered valid (the “gold standard”). If there is a high correlation, this gives a good indication that the test measures what is intended. Alternatively, measures that should not be related, should demonstrate low correlations, therefore providing evidence for discriminant validity of the measure.

#### Assessment Procedure

In addition to its psychometric robustness, the suitability for a stress measure for practical and research purposes can be defined by a number of practical and procedural attributes of the instrument. These include the length of the assessment (determined by the number of the items and procedure), the presentation format (paper/pencil, digital, oral), the role of the assessor (group, guided or individual assessment), and the intended population. These attributes define the context and organizational prerequisites for administration and whether it should be stipulated in the manual.

#### Suitability for Adults With MID

##### Review of the Literature

One of the main purposes of this review concerned investigating the suitability of the measure for people with MID. After analyzing each stress self-report measure, we performed a literature search to see if any scientific evidence could be found on the use of the self-report measure in populations that included people with MID. The search was performed in Google Scholar. The following search string was used to guide the search: “learning disabilit^*^” OR “developmental” OR “mental retard^*^” OR “intellectual dis^*^” AND [self-report measure]. If a reference was made regarding the suitability of the particular self-report measure in people with intellectual disabilities, learning disabilities, or developmental problems, we reported this.

##### Expert Consultation

As mentioned, adaptations to standard self-report instruments are generally needed to make them suitable for people with MID. As yet, no comprehensive guidance on how to make these adaptations is available (Kooijmans et al., 2021a). To be able to provide more information, we consulted experts in the field of MID research and clinical practice. We used purposive sampling to select internationally renowned researchers in the field of intellectual disability research. The sampling frame was devised from a previously conducted systematic review (Kooijmans et al., 2021a). This sample was expanded by probing the authors' network and asking colleagues in the field of ID research to nominate researchers and clinicians they deemed experts on the topic. We then invited 40 experts from the United States, Europe and Australia to complete an online survey. Of these, 13 experts (33%) from four European countries completed the survey. Participants were academic and clinical staff from the United Kingdom, the Netherlands, Belgium, and Germany with considerable experience in working and conducting research with people with (M)ID. See Table 1 for an overview of the characteristics of the participating experts.

**Table 1:** Demographic characteristics of experts consulted.

**Total *N* = 13**	*n* (%^*^)
**Country of residence**	
The Netherlands	6 (46%)
United Kingdom	5 (38%)
Germany	1 (8%)
Belgium	1 (8%)
**Current employment**	
Academic setting	9 (69%)
Clinical setting	2 (15%)
Joint academic / clinical	2 (15%)
**Years of experience working with people with MID**	
1–5	2 (15%)
6–10	4 (31%)
11–20	3 (23%)
20+	4 (31%)

* *Percentages not adding up to 100% due to rounding differences*.

In the survey, the experts were asked to reply to open-ended questions on the subject of how to attune self-report measures to the needs and abilities of people with MID. They were asked to forward suggestions that address the content of self-report stress measures, such as language, response options and supportive media, and procedural issues, such as assessment procedures, questionnaire structure, and instructions. Thematic analysis was applied to synthesize the results into general recommendations. In further Delphi rounds we explored which of the forwarded recommendations were endorsed by the majority of experts. The expert consultation on self-report stress measures was carried out within the context of a larger Delphi study on self-report instruments for persons with (M)ID. A more detailed description of the methodology applied is provided in the Delphi study research article that has been submitted for publication (Kooijmans et al., 2021b). As part of the assessment of the suitability of the included self-report stress measures for people with MID, we compared the recommendations from the survey with the published information of the self-report stress measures. The recommendations that were endorsed by the experts and the performance of each measure on each recommendation are presented in **Table 3**.

## Results

A total of 13 stress-related self-report measures met the final inclusion criteria. Nine of these explicitly focus on stress as an outcome and four on state anxiety as an outcome. Of the included self-report stress measures, the Glasgow Anxiety Scale for Intellectual Disabilities (GAS-ID), the Life Inventory (LI), and the Self-Rating Anxiety Scale for Intellectual Disabilities (SAS-ID) were specifically developed for assessing stress in adults with (mild) intellectual disabilities. First, we share our findings of the analysis of the self-report stress measures included purely from the perspective of the literature. We then discuss the findings of experts consulted, and present the integration of both types of data in **Table 3**.

### Included Self-Report Stress Measures

The characteristics of each individual instrument are described for each of the three criteria: psychometric quality, assessment procedure, and suitability for people with MID based on the consultation of experts and the scientific literature. The findings are summarized in Table 2 and described in more detail below for each instrument (in alphabetical order).

**Table 2:** Included self-report stress measures.

**Title (author/s, publication date)**	**Outcome**	**Target group**	**Psychometric quality:** **(a) independent validation research available?** **(b) number of available validating studies (approximately)** (c) internal validity (Cronbach's α)*	**Design:** **a) number of items** **b) response options** **c) duration of administration**	**Published information on applicability with people with MID available?**
Beck Anxiety Inventory (BAI), Beck et al. (1988)	State anxiety	Adults	a) yes b) >10 c) average across studies = 0.91	a) 21 items b) 4-point Likert scale c) 10 min max.	Yes Adaptation for MID by Lindsay and Skene (2007)
Depression Anxiety Scales (DASS) (Lovibond and Lovibond, 1995b)	Stress + state anxiety	Adults	a) Yes b) >20 c) α of 0.84–0.92 for DASS-Anxiety, and 0.90 to 0.95 for DASS-Stress	a) 21 short form, 42 regular form b) 4-point Likert scale c) 5–10 min short form, 10–20 min long form (all three subscales)	No Parkitny and McAuley (2010): “certain patient groups (e.g., the developmentally delayed…) may have difficulty understanding the questionnaire items or responding to them in an unbiased manner.
Derogatis Stress Profile (DSP) (Derogatis, 1987)	Stress	Adults	a) yes (only 1 study found) b) 2 c) α between 0.83 and 0.88 for different domains	a) 77 items b) 5-point Likert scale + VAS (0–100) for subjective stress experience c) 12–15 min	No
Glasgow Anxiety Scale (GAS-ID) (Mindham and Espie, 2003)	State anxiety	“People with an intellectual disability” (age/level of ID not specified)	a) Yes (only 1!) b) 2 c) >0.80	a) 27 b) 3-point Likert scale of frequency c) 5–10 min	Yes
Index of Clinical Stress (ICS) (Abell, 1991)	Stress	Adults and youths age 12+; Reading level > grade 4	a) No b) 2 (developer + affiliated researchers) c) 0.96 (Abell, 1991)/0.90 (Hudson et al., 1995)	a) 25 b) 7-point Likert scale of frequency c) not specified, but stated as “rapid.”	Yes Manual: “Persons who are only mildly impaired might be able to complete the WAS scales with considerable accuracy. The major things to watch for are the literacy skills, cognitive development, and ability to integrate affective responses with the item content and meaning of each of the scales.” Flesch reading ease: 89 (6th grade level); Gunning Fog Index: 6 (sixth grade level); Flesch-Kincaid Grade Level: 4.
Lifestress Inventory (LI) (Bramston and Fogarty, n.d.)	Stress	Age not specified; “suitable for administration to a wide range of people, including the mildly intellectually handicapped”	a) No b) 3 c) 0.80	a) 30 b) 4-point Likert scale + visual aid showing a series of buckets empty through to full can be used to improve understanding of the Likert-type options c) not specified	Yes
Psychological Stress Measure (PSM-9; Lemyre and Tessier, 1988)	Stress	Adults	a) no b) 4 c) 0.89	a) 9 b) 8-point Likert scale c) not specified	No
Perceived Stress Questionnaire (PSQ; Levenstein et al., 1993)	Stress	Adults	a) yes b) 6 c) ranging from 0.90 to 0.93	a) 30 regular, 20 short form b) 4-point Likert scale c) 5 min	No
Perceived Stress Scale (PSS; Cohen et al., 1983)	Stress	Adults (“community samples with at least a junior high education”; “accessible to any subpop-ulation”)	a) Yes b) 19 or more c) above 0.70 across studies	a) 10 b) 5-point Likert scale c) 5–10 min	No
Stress Arousal Checklist (SACL; Cox and Mackay, 1985)	Stress	Not specified	a) Yes b) 6 or more c) for stress scale ranging from 0.81 to 0.86, lower for arousal scale	a) 30 b) 4-point Likert scale c) not specified	No
Self-Rating Anxiety Scale for adults with Intellectual Disabilities (SAS-ID; Lindsay and Michie, 1988) Adaptation of SAS (Zung, 1971)	State anxiety	People with an intellectual disability (age/level of ID not specified)	a) Yes b) 3 or more c) average of 0.80 across studies	a) 20 b) yes-no answer format. c) 5–10 min	a) Yes b) Adaptations from the original instrument include yes–no response format, rewording of the items, and addition of supplementary items.
SOS Stress Overload Scale (Amirkhan, 2012)	Stress	Adults	a) No b) 3 c) 0.94	a) 30 or 10-item short form b) 5-point Likert scale c) not specified	No
STAI State-Trait Anxiety Inventory (Spielberger, 1973)	State anxiety	Adults (used in ID research)	a) yes b) lots of studies in many different languages c) ranges from good to excellent across several populations	a) 20 items for the State scale (Y1 form) b) 4-point Likert scale. c) ~10 min for “less educated or emotionally disturbed persons.”	a) Yes: Manual specifies some instructions for the assessor in the case of guided assessment. b) Norms are based on a sample of “working persons,” which generally will include relatively few persons with MID. c) The STAI is frequently used in research with persons with MID in unaltered form.

*References and additional psychometric results are provided in detailed descriptions of the single measures in the result section*.

#### Beck Anxiety Inventory (BAI)

The original publication of the BAI dates back to 1988 (Beck et al., 1988) and it is still widely used today. It measures (state) anxiety symptoms and their level of intensity over the past week. It includes 21 items that target both somatic and more cognitive symptoms of state anxiety, for which respondents rate the intensity on a 4-point rating scale ranging from “not all” to “severely.” The total score is rated as minimal, mild, moderate or severe (state) anxiety.

##### Psychometric Quality

The BAI was found to have high internal consistency (average α coefficients across studies = 0.91; Bardhoshi et al., 2016) and adequate test-retest reliability (test–retest reliability = 0.65; Bardhoshi et al., 2016). It demonstrated both convergent validity with related measures of anxiety (other self-report instruments, diaries, clinical ratings; correlation coefficients ranging from 0.24 to 0.81; Bardhoshi et al., 2016) and moderate discriminant validity with other types of psychopathology (e.g., non-significant correlations with a measure of OCD symptomatology; Williams et al., 2013; moderate correlations with the Beck Depression Inventory; average *r* of 0.59 across studies; Bardhoshi et al., 2016). Both exploratory and confirmatory factor analytic studies generally support a two-factor structure in clinical populations. One factor represents cognitive symptoms of anxiety and the other represents somatic symptoms (Wilson et al., 1999).

##### Assessment Procedures

The BAI can be self-reported or interviewer-administered. Self-report generally takes a maximum of 10 min to complete. It can be administered in paper-and-pencil or interview format, but it is also available online.

##### Suitability for Adults With MID

The factor structure and other psychometric properties of the BAI were examined in a sample of people with MID (*N* = 108; Mean IQ 67.1; Lindsay and Skene, 2007). To ensure that most people in the sample were able to meaningfully complete the BAI, some adaptations were made. The terminology of some of the items was simplified and the four-point response scale was presented in the form of four bar graph histograms of differing sizes. All questions were read aloud to all respondents by the assessor. On the basis of the analyses in their study, Lindsay and Skene (2007) asserted that people with MID appear to use the BAI reliably and consistently, and that the factors emerging from the sample were similar to those from mainstream populations.

#### Depression Anxiety Stress Scales (DASS)

The DASS (Lovibond and Lovibond, 1995a) measures three emotional states: depression, anxiety and stress. Three subscale scores for each of the emotional states are obtained that can be compared to norms and clinical cut-offs. For the purpose of this review, the properties of the Stress subscale were considered.

##### Psychometric Quality

High internal consistency coefficients are reported for each of the subscales of the 42-item and the 21-item versions (Cronbach's α of 0.90–0.95 for DASS-Stress; Parkitny and McAuley, 2010). Good evidence has been found for the construct validity through factor analyses (Lovibond and Lovibond, 1995a; Crawford and Henry, 2003) and convergent validity for the anxiety subscales of both the long and short versions of the DASS (correlation between DASS and BAI *r* = 0.81; Lovibond and Lovibond, 1995a), but the properties of the stress subscale have been evaluated less extensively. Research in clinical populations has demonstrated responsiveness to treatment effects in, among others, psychiatric patients (Lovibond and Lovibond, 1995a; Ng et al., 2007) and persons with autistic spectrum disorders (Park et al., 2020).

##### Assessment Procedures

According to the manual (Lovibond and Lovibond, 1995b), completion takes 10–20 min for the 42-item version that comprises all three subscales. The shorter 21-item version of the DASS (DASS-21) takes 5–10 min to complete. A respondent indicates to what extent the statements applied to their lives over the past week on a 4-point scale. The DASS can be administered by paper-and-pencil or computer. The paper-and-pencil questionnaires and scoring forms are available at no cost from the developers' website. No specific training is needed to administer and score the DASS. Numerous officially endorsed translations of the DASS are available in many languages.

##### Suitability for Adults With MID

No empirical studies involving people with MID were found. Generally, people with MID were excluded from psychometric studies. The developers state that the DASS should not be presumed valid for some subpopulations, including “[persons with]…low literacy…” (Psychology Foundation of Australia, 2021). This effectively precludes many people with MID from using the DASS.

#### Derogatis Stress Profile (DSP)

The DSP is a self-report inventory rooted in interactional stress theory (Derogatis, 1987). Assessment of the DSP results in a detailed profile that identifies stressors on an environmental, personality, and emotional level, in interaction with each other. Cumulative scores provide a quantitative overall summary estimate (global stress score) of the respondent's current stress level.

##### Psychometric Quality

Strong support for the internal consistency (Cronbach's α > 0.80 for all “stress domains”), reliability (test-retest coefficients > 0.72 for subscales and total scores) and validity of the DSP (by means of factor analyses) is provided in a small clinical sample and a larger non-clinical sample (Derogatis, 1987). A study on the correlation between several associated stress measures, including physiological correlates, yielded some support for the convergent and construct validity of the DSP (Dobkin et al., 1991).

##### Assessment Procedures

Respondents are asked to rate 77 statements on a 5-point scale ranging from “not-at-all true of me” to “extremely true of me.” According to the information provided on the developer's website, “the scale takes ~12–13 min to complete under normal conditions, although some individuals may require a few minutes longer” (Derogatis Testing, 2021).

##### Suitability for Adults With MID

No empirical studies addressing the suitability of the DSP for people with MID were found. The number of items and the complexity of the measure suggest that assessment may be a challenge for most people with MID (Hartley and MacLean, 2006; Bell et al., 2018).

#### Glasgow Anxiety Scale for People With an Intellectual Disability (GAS-ID)

The GAS-ID (Mindham and Espie, 2003) was specifically developed for people with (M)ID to provide a reliable measure of state anxiety. It targets cognitive and emotional symptoms of state anxiety in the past week, as well as physiological symptoms that are assessed in the here and now.

##### Psychometric Quality

The GAS-ID showed sufficient methodological quality and excellent reliability (Cronbach's α = 0.96; test-retest *r* = 0.95) and validity results (ρ correlation coefficient of 0.75 with the BAI; ρ = 0.52 with pulse rate) as reported by the developers themselves (Mindham and Espie, 2003). However, only one external validation study was found (Hermans et al., 2013); the authors concluded that the GAS-ID can be regarded as a reliable self-report measure. High Cronbach's α's (>0.80) and test-retest ICC (0.89) were reported, and the GAS-ID showed satisfactory correlations with related measures (correlation with the HADS-A of *r* = 0.61).

##### Assessment Procedures

No manual is available for the GAS-ID. The assessment time is reported to be 5–10 min (Mindham and Espie, 2003). The questionnaire is administered as a structured interview. Respondents are asked to rate how often they experienced 27 expressions of fears, worries and physiological symptoms in the past week on a 3-point answer scale (from “never” to “always”). Furthermore, respondents are asked whether they experience any physiological symptoms associated with state anxiety in the here and now. Clinical cut-off scores are proposed by Mindham and Espie (2003), but they state that more research is needed.

##### Suitability for Adults With MID

The GAS-ID is designed specifically to be administered to people with MID. In the process of development, several alternative versions were tested for optimum suitability for people with MID. The resulting measure is perceived by the authors as being suitable for use with those people with MID who demonstrate sufficient ability to communicate verbally in day-to-day interactions (Mindham and Espie, 2003). The GAS-ID is frequently used in research on stress and anxiety with people with MID (e.g., Hartley and MacLean, 2008), is referenced as a preferred diagnostic tool in clinical guidelines for people with MID (e.g., Davis et al., 2008), and is mentioned in several textbooks on diagnostics and treatment of people with (M)ID (e.g., Stavrakaki and Lunsky, 2007).

#### Index of Clinical Stress (ICS)

The ICS (Abell, 1991) is a self-report questionnaire for individuals older than 12 years. It measures the degree or magnitude of clients' perceptions of personal stress, which is defined by a “… perceived imbalance between the demands of daily living and a person's ability to respond.” The ICS is part of the Walmyr Assessment Scales (WAS), a compendium of more than 25 short-form measurement scales designed for use in assessing the severity or magnitude of a variety of personal and social problems (Walmyr Publishing Company, 2021).

##### Psychometric Quality

Psychometric evaluation studies were conducted by the developer or researchers affiliated to the WAS (Abell, 1991; Hudson et al., 1995). High Cronbach's α's of 0.96 (Abell, 1991) and 0.90 (Hudson et al., 1995) were reported. Evidence for convergent validity was demonstrated by means of significant correlations with associated constructs (mean *r* = 0.48) and nonsignificant correlations with discriminant factors (mean *r* = 0.08).

##### Assessment Procedures

The respondent is required to respond to the 25 items on the test form by selecting one response from a 7-point scale ranging from “none of the time” to “all of the time.” The respondent is expected to fill in the questionnaire unassisted. The WAS manual details no administration times, but is reported to be “rapid.” The ICS is available in paper-and-pencil form and can be administered digitally through the publisher's own digital administration application.

##### Suitability for Adults With MID

The manual states that those completing the questionnaire must be literate and have no severe cognitive impairment. Readability statistics for the measure are given. The Flesch-Kincaid Grade Level of four suggests that a fourth level reading grade is required to complete the form autonomously. As the majority of people with MID are unable to read beyond grade three level (Conners, 2003), autonomous completion of the ICS may be challenging for many. However, the ICS was developed for individuals from the age of 12 years upwards, meaning that the level of understanding may be appropriate for some people with MID.

#### Lifestress Inventory (LI)

The LI (Fogarty et al., 1997) is a self-report questionnaire designed to measure frequency and impact of stressors in daily life. It was developed specifically for people with MID as an update of the Subjective Stress Scale (SSS) that is no longer available.

##### Psychometric Quality

In three studies, none of which were conducted by independent authors, the psychometric quality was found to be sufficient (Fogarty et al., 1997; Bramston et al., 1999; Lunsky and Bramston, 2006). For internal consistency, Lunsky and Bramston (2006) found Cronbach's α to equal 0.80. In the same study, some evidence was presented for the convergent validity of the LI, by showing significant correlations with related measures (*r* = 0.64 to 0.78). Modest correlations were presented between self-report and informant measures (*r* = 0.34 to 0.70). According to Fogarty et al. (1997), confirmatory factor analysis indicated three underlying factors that impact the experience of stress in daily life. These factors were labeled General Worry, Negative Interpersonal Relations, and Coping.

##### Assessment Procedures

According to the scoring instructions / manual provided by the authors (Bramston and Fogarty, n.d.), the 30 items of the LI are intended to be read aloud. A series of buckets from empty to full can be used as a visual representation for the response options to facilitate understanding. Other possibilities to ensure that an item is understood correctly include repeating or re-wording a question, as well as asking the respondent to elaborate on their answer to make sure they interpreted the question correctly. As an extra response option, “0” indicates that an item/event was not experienced by the respondent; this option helps establish a frequency score. The other response options—from 1 (“no stress”) to 4 (“a great deal of stress”)—indicate the impact of single stressors. Assessment is preferably completed by an experienced psychologist.

##### Suitability for Adults With MID

The LI has been specifically developed for people with MID and research into validation has been, as quoted above, carried out with people with MID. Notably, the LI was developed by means of focus groups with people with MID and staff members, and was designed to be easily understood and completed by people with MID (Scott and Havercamp, 2018).

#### Perceived Stress Scale (PSS)

The PSS (Cohen et al., 1983) has become one of the most widely used psychological instruments to measure the degree to which situations in people's lives are appraised as stressful. Cohen et al. (1983) define psychological stress as the extent to which a person perceives that demands exceed his/her ability to cope.

##### Psychometric Quality

Although scores on the 14-item PSS exhibit good reliability estimates across the literature, four of the items tend to perform poorly when evaluated using exploratory factor analysis (Cohen and Williamson, 1988; Lee, 2012). As a result, the PSS is commonly implemented using the 10-item form. In the review of Lee (2012) on the psychometric qualities of the PSS, it is shown that all included studies (*N* = 19) reported α coefficients of > 0.70. The test-retest reliability of the PSS-10 was assessed in four studies, and met the criterion of > 0.70 in all cases. The PSS correlated significantly and predictably with a range of other measures of stress and pathology (correlations typically in the 0.30–0.70 range), such as the Job Responsibilities Scale, HADS, and STAI. Additionally, higher PSS scores have been shown to be associated with higher levels of cortisol; a biological indicator of stress (van Eck and Nicolson, 1994).

##### Assessment Procedures

The PSS is available in a 14 and 10-item form and the average completion time is 5–10 min. Items are designed to tap how unpredictable, uncontrollable, and overloaded respondents generally find their lives. The scale also includes a number of direct queries about current levels of experienced stress.

##### Suitability for Adults With MID

The PSS is designed for use in community samples for those with at least a junior high school education. Although there is no information available on the use of the PSS in adults with MID, some research has been carried out with younger college students with disabilities, such as learning disabilities, ADHD, and autism spectrum disorders (Janusis and Weyandt, 2010). The students with disabilities tended to score higher on the PSS, but the differences did not approach significance. No separate norms for people with disabilities were constructed on the basis of this study.

#### Perceived Stress Questionnaire (PSQ)

The PSQ (Levenstein et al., 1993) measures the experience or perception of stress by the individual during stressful situations, and is considered valid in the context of a transactional model of stress (Kocalevent et al., 2007). The PSQ was developed for use within the field of clinical psychosomatic research (Levenstein et al., 1993, 2000). There are two forms of the PSQ: the “general” (the last 2 years) and the “recent” (during the last 4 weeks) form.

##### Psychometric Quality

The original authors developed the instrument in English and Italian and validated it among 230 subjects (Levenstein et al., 1993). Internal consistency of the original English version (measured by Cronbach's α) ranges from 0.80 to 0.86 (Levenstein et al., 1993; Kocalevent et al., 2007), and research on test-retest reliability (Pearson correlation coefficients *r* between 0.80 and 0.86; Levenstein et al., 1993; Sanz-Carrillo et al., 2002). The PSQ shows positive associations with compatible self-report measures such as Cohen et al. (1983) Perceived Stress Scale (PSS; *r* = 0.73; Levenstein et al., 1993). Notably, there are some indications that PSQ scores seem to differ between populations of patients and healthy individuals, and that scores seem to be sensitive to change after treatment (Fliege et al., 2005).

##### Assessment Procedures

The PSQ has 30 items. Response options and items of both the PSQ-General (past 1–2 years) and the PSQ-recent (past month) are identical. Respondents are asked to estimate how often they deal with stress-related experiences on a 4-point Likert scale. While no extensive manual has been published, free scoring instructions are available to researchers. The administration time is expected to be 5 min. Translations along with validation studies are available in Swedish (Rönnlund et al., 2015), Norwegian (Østerås et al., 2018), Spanish, Chinese, and German. The instrument is available at no cost under a Creative Commons license.

##### Suitability for Adults With MID

No information on the suitability for people with MID has been found in previous empirical studies. The PSQ was originally intended for adults, but has also been successfully validated for adolescents aged 15–16 years (Østerås et al., 2018). Mutz and Müller (2016) used the PSQ to assess 14-year-old German upper secondary school pupils, without commenting on the application of the instrument to the target group. The adolescent research projects indicate that research about the usefulness of the instrument for (some) people with MID can be recommended.

#### Psychological Stress Measure (PSM-9)

The PSM was first published in 1988 (Lemyre and Tessier, 1988) and updated in 2003. The PSM-9 is an abridged nine-item version of the original 49-item assessment of self-reported state stress. Respondents are asked to rate stress symptoms they experienced in the past 3–4 days on an 8-point Likert scale (from “not at all” to “extremely”). The result is a single-factor indicator of perceived stress.

##### Psychometric Quality

The authors report a wide range of reliability (Cronbach's α's > 0.90; test-retest *r*'s 0.68–0.80) and validity coefficients for the 49-item version in a series of publications by the developers of the instrument (Lemyre and Tessier, 1988, 2003; Lemyre et al., 1990, 2009). The psychometric properties of the short PSM-9 version are reported to be “the same as the original version” (Lemyre and Tessier, 2003), but only a Cronbach's α of 0.89 is reported for the PSM-9. No external validation studies have been published.

##### Assessment Procedures

The PSM-9 appears to be a short single-page paper-and-pencil questionnaire. No scoring instructions could be retrieved. Only a French version of the manual was published (Lemyre et al., 1990), but it could not be retrieved by the reviewers.

##### Suitability for Adults With ID

No evidence was found that the PSM-9 would be suitable for people with MID.

#### Self-Rating Anxiety Scale for Intellectual Disabilities (SAS-ID)

The SAS-ID is an adaptation of the Zung Self-Rating Anxiety Scale for persons with ID by Lindsay and Michie (1988). The SAS is a 20-item self-report assessment instrument for measuring state anxiety. Respondents are asked to indicate to what extent a series of statements apply to themselves within a period of 1 or 2 weeks prior to assessment. A total score reflects a general level of state anxiety as experienced by the respondent.

##### Psychometric Quality

Several researchers have assessed the psychometric quality of the SAS-ID (Lindsay et al., 1994; Masi et al., 2002; Ramirez and Lukenbill, 2008). Psychometric evaluation was conducted by independent researchers and those affiliated to the original developers. Internal consistency coefficients (Cronbach's α) averaged a satisfactory 0.80. Convergent validity was established by finding significant correlations between the SAS-ID and related self-report instruments and diagnostic interviews (correlation coefficients ranging from 0.33 to 0.73).

##### Assessment Procedures

The SAS-ID is a 20-item scale with a yes–no response format. It takes 5–10 min to complete. The SAS-ID is presented to respondents orally on an individual basis. Assessors are instructed to rephrase or reword the questions if the respondents appear to lack understanding.

##### Suitability for Adults With ID

The SAS-ID is an adaptation of the original SAS that is intended for use in the general population. Adaptations are made to ensure that most people with MID are able to meaningfully complete the assessment with assistance. Adaptations made to the original are the use of a yes-no response format instead of a 4-point Likert-type scale and the rewording of items perceived to be difficult. The SAS-ID has occasionally been used in research involving people with MID (e.g., Carraro and Gobbi, 2012) and is mentioned in textbooks on diagnostics and treatment of persons with (M)ID (e.g., Hatton and Taylor, 2013; Vargas-Vargas et al., 2019).

#### State-Trait Anxiety Inventory, State Version (STAI-S)

The state version of the STAI (Spielberger, 1973) is one of the most long-standing and commonly used clinical self-rating scales to measure state-anxiety, which is defined as a temporal cross section in a person's emotional stream of life, consisting of subjective feelings of stress, tension, apprehension, nervousness, worry, and activation of the autonomic nervous system (Cattell and Scheier, 1961; Spielberger, 1973). In research, the 20-item STAI subscale is often used to measure state-anxiety before and after an intervention or task. Translated forms of the STAI are now available in more than 60 languages (Spielberger and Reheiser, 2009).

##### Psychometric Quality

Many psychometric evaluation studies have been published which show that the STAI-S provides excellent psychometric properties: the internal consistency measured using Cronbach‘s α coefficient ranges from good to excellent (i.e., > 0.70) across several populations (e.g., Spielberger, 1973; Creamer et al., 1995; Fonseca-Pedrero et al., 2012; Ortuno-Sierra et al., 2016). Noteworthy, α coefficients are typically higher for the STAI-S when state anxiety is assessed under conditions of psychological stress (Spielberger, 1973; Spielberger and Reheiser, 2009).

##### Assessment Procedures

The STAI-S is a 20-item self-rating inventory which may be given either individually or to groups. The scale is composed of short verbal statements that participants have to rate using a 4-point Likert scale according to the subjective experienced intensity of each described feeling (1 = not at all, 4 = very much so). It is clear that the questionnaire's ease of administration, as well as the simple and straightforward scoring procedure have led many researchers to use this specific instrument (Rossi and Pourtois, 2012).

##### Suitability for Adults With ID

Although no studies have been published on the applicability of the STAI-S in persons with (M)ID, a STAI child-version (STAI-C) has been developed (Spielberger, 1973), especially constructed for 9–12-year old children. The STAI-C manual states that the scale may also be used with older children/adolescents who are below average in ability. In future research, the appropriateness of the STAI-C version for use in people with MID should be investigated.

#### Stress Arousal Checklist (SACL)

The SACL (Mackay et al., 1978) is a list of mood adjectives intended to measure stress experience as well as arousal. The authors refer back to work by Thayer (1967) and his factor analysis of the Activation-Deactivation Adjective List (AD-ACL). The two-dimensional structure of stress and arousal is explained as follows: “The stress dimension refers to the perceived favorability of the external environment, while arousal refers to ongoing autonomic and somatic activity” (Cox and Mackay, 1985).

##### Psychometric Quality

In an independent factor analysis, the two-factor structure found by the original authors has been replicated (McCormick et al., 1985). This study also supports the two-dimensional model of stress and arousal operationalized in the SACL. Reliability was found to be relatively high in several studies (>0.70), especially for the stress scale, while α coefficients showed more variance for the arousal scale (Watts et al., 1983). Evidence for the construct validity of the SACL was found in factor analyses (King et al., 1983; Fischer and Donatelli, 1987; Fischer et al., 1988). However, Hinton et al. (1991) stated that in their view, the stress scale of the SACL does not measure stress as defined by the authors and “is virtually identical to the state version of the STAI.”

##### Assessment Procedures

There does not seem to be a published manual, but the authors provide scoring instructions and note that “scoring keys are easily made” (Cox and Mackay, 1985, p. 284). The 30-item list consists of positive and negative adjectives, for each of which the symbols “++,” “+,” “?,” or “-” can be circled by respondents. Responses can be summed up separately for the “stress” and “arousal” subscales (Cox and Mackay, 1985, p. 284).

##### Suitability for Adults With MID

No empirical evidence was found for the suitability of the SACL for people with MID.

#### Stress Overload Scale (SOS)

The SOS (Amirkhan, 2012) is designed to measure “stress overload,” a state described in stress theories as occurring when demands overwhelm resources. Respondents are asked to answer 30 questions and reflect on the occurrence of stress-related feelings and cognitions in the past week. Total scale scores and scores on two subscales—Personal Vulnerability and Event Load—are calculated. A short 10-item version (the SOS-S) is also available (Amirkhan, 2018).

##### Psychometric Quality

All psychometric evaluation studies were conducted by the developers (Amirkhan, 2012, 2018; Amirkhan et al., 2015). They report an excellent internal consistency of the SOS (with Cronbach's α's > 0.94 for both subscales and the measure as a whole). Test-retest coefficients averaged 0.75 over a 1 week period. Convergent validity has been demonstrated in significant correlations with other measures of stress (e.g., correlation coefficient *r* of 0.45 with the PSS-10) and criterion validity has been shown in the SOS's ability to predict illness following a stressful event. Psychometric properties for the original and short versions are all but identical.

##### Assessment Procedures

Participants are asked to rate feelings and cognitions related to life stress on a 5-point Likert scale (from “not at all” to “a lot”). No information on the duration of the assessment of the original or short forms has been published and no manual is available. Scoring instructions are attached to the form.

##### Suitability for Adults With ID

The development and validation of the SOS made use of community samples. Some attention was paid to make sure that “… Only items that were consistently understood across [a] wide socioeconomic and ethnic spectrum were chosen for the SOS” (Amirkhan, 2012). However, its comprehensibility and general usefulness for people with MID has not yet been demonstrated.

### Results of the Expert Consultation

The experts were asked to reply to open-ended questions on the subject of how to attune self-report measures to the needs and abilities of people with MID. They unequivocally indicated that the factors that improve appropriate use by people with MID in general also apply to the self-reported measurement of stress. Thematic analysis of the answers revealed six general recommendations relevant to the measurement of stress in people with MID.

The first recommendation was to use concrete and easy-to-understand vocabulary, simple grammar, and short sentences. The next was to use relatively short time frames for the retrieval of information. Assessors should not ask to retrieve information over longer periods than 1 week, as time processing abilities are generally impaired. A third recommendation relates to the use of Likert scales. When designing self-report measures for people with MID, the number of response options in Likert scales should be limited to three for people with moderate ID to MID and five to people with MID to borderline intellectual functioning. Fourth, an “I don't know” option should be included in both forced-response and open-ended questions to prevent invalid answers from those who do not understand the question. A fifth recommendation was to use visualizations to support the meaning of questions and responses, although how exactly these should be configured was not specified. In regard to the assessment procedures, a sixth recommendation was to use pre-scripted alternative wording if the respondent seems unable to understand the question. Standardization ensures comparability of scores across assessments. The extent to which these factors were reflected in the self-report measures' design and assessment procedures differed across the included instruments. An overview of the suitability of each self-report stress measure for people with MID, according to the experts, is presented in Table 3.

**Table 3:** Factors that determine the suitability of included self-report stress measures for people with MID according to the expert consultation.

	**Use of easy-to-under-stand language**	**Max. 1-week time frame**	Max. 5 answer options*	**“I don't know” answer option**	**Use of visual support**	**Scripted alternative wording**
BAI	✓	✓	✓	**X**	✓	**X**
	(adaptation by Lindsay and Skene, 2007)				(adaptation by Lindsay and Skene, 2007)	
DASS	**X**	✓	✓	**X**	**X**	**X**
DSP	**X**	✓	✓	**X**	**X**	**X**
GAS-ID	✓	✓	✓	**X**	**X**	**X**
ICS	✓	✓	**X**	**X**	**X**	**X**
LI	✓	/	✓	**X**	✓	**X**
PSM-9	/	✓	**X**	**X**	**X**	**X**
PSQ	/	**X**	✓	**X**	**X**	**X**
PSS	/	**X**	✓	**X**	**X**	**X**
SACL	/	✓	✓	**X**	**X**	**X**
SAS-ID	✓	✓	✓	**X**	**X**	**X**
SOS	/	✓	✓	**X**	**X**	**X**
STAI-S	/	✓	✓	**X**	**X**	**X**

## Discussion

The need to measure the degree of stress as accurately as possible in people with MID is reflected in both the literature reviewed and the information of the consulted experts. This can be seen as a response to the fact that people with MID are much more vulnerable to stress (Hatton and Emerson, 2004; Scott and Havercamp, 2014). Persistent stress experiences in people with MID may lead to more impaired information processing (Heyman and Hauser-Cram, 2015) which will adversely affect coping skills. Our study not only provides the first overview and analysis of self-report stress measures, but also provides more insights in how self-report stress measures can be adequately attuned to the needs of people with MID. Of the 13 self-report stress measures found, three measures were specifically designed for use with adults with (M)ID. For the remaining 10 measures, no empirical support for use in these populations was found and further investigation is warranted.

### Main Findings

The Lifestress Inventory (LI) was specifically designed for the MID population. Two others, the Glasgow Anxiety Scale for people with Intellectual Disability (GAS-ID) and the Self-rating Anxiety Scale for adults with Intellectual Disabilities (SAS-ID) reported that they were fit for use with people with ID, but the user manuals did not specify the exact intelligence range. As the items concerned mainly refer to insights, feelings and experiences from daily life, participants must be able to grasp these abstract concepts, translate them to their everyday experiences and formulate a meaningful response. This suggests that they are targeted toward adults with MID instead of the total ID population. Generally, these three self-report stress measures have in common that they use items that require a response on simple Likert scales, which could possibly be combined with visual representations of answer alternatives. This is in line with findings reported in previous studies as well as the expert consultations in our study, which show agreement that responses requiring a simple Likert rating scale or only yes/no can lead to appropriate responses from individuals with MID (Heal and Sigelman, 1995; Ramirez, 2005; Hartley and MacLean, 2006). For those individuals in the lower range of MID, pictorial representations of response alternatives could increase the likelihood of gaining appropriate responses (Hartley and MacLean, 2006), which was echoed by the experts consulted.

Despite the lack of empirical support, our findings also show that some of the other stress self-report measures seem to be more or less suitable for adults with MID. First, some evidence was provided in previous validity studies on populations in which participants with intellectual, learning, or developmental disabilities were also included. This applies to the BAI (see Lindsay and Skene, 2007), the DASS (see Psychology Foundation of Australia, 2021), and the PSS (see Janusis and Weyandt, 2010). Second, other self-report stress measures stated that they could also be used in younger aged populations, which may suggest that, at least in terms of comprehensibility, they may be suitable for people with MID. This applies to the STAI-child version (9–12 years), the ICS (from 12 years), and the PSQ (from 14 years). Hurley (2008) suggests that the use of instruments designed for children may offer a useful basis for adaptation, because the measures use concrete levels of vocabulary and simple sentence structures. This process has also been used by many other researchers (e.g., Marshall and Willoughby-Booth, 2007; Guerin et al., 2009). However, since these stress self-report measures have not been validated specifically for the adult MID population, we recommend thoroughly screening the measurement construct and assessment procedure before using them in clinical practice or in future research (Kooijmans et al., 2021a).

The findings from the expert consultations show the importance of adding an extra “I don't know” answer alternative to prevent people with MID who do not understand the question from filling in a random answer (Bell et al., 2018). However, none of the self-report stress measures, even those specifically developed for people with MID, included this option. The Lifestress Inventory (LI) added the answer alternative “actually not experienced,” but this refers to the fact that the participant did not experience any stress at all. In addition, none of the self-report measures included “alternative wording” to the questions and/or answer alternatives, as recommended by the expert panel. On the other hand, helping factors such as allowing assessment assistance (SAS-ID) or having someone else read the items (LI and GAS-ID) were not mentioned by any of the experts. Finally, response visualizations seem to be missing from both the GAS-ID and SAS-ID. This is remarkable, as this is considered one of the most important factors with regard to suitability for people with MID, both in the literature and by the experts consulted (e.g., Hartley and MacLean, 2006; Scott and Havercamp, 2018). Of the three self-report stress measures for people with MID, the LI appears to be most consistent with the findings of the experts. However, our findings show that in addition to consulting experts, screening the assessment procedures of existing self-report measures specifically adapted or designed for people with (M)ID is a worthwhile exercise.

### The Added Value of Self-Reported Information

Although proxy reports are commonly used in MID, self-report measures prove to be more accurate and more sensitive, even in the MID population (Moss et al., 1996; Scott and Havercamp, 2018). The importance of obtaining self-reported information on subjective stress experiences of people with MID is also reflected in the increased recognition in our society that people with (M)ID are full citizens with the same rights as non-disabled persons, meaning that participation and social inclusion should dominate all organized activities (e.g., Devi, 2014; Giesbers et al., 2019). In other words, including the opinions, feelings, and thoughts of people with MID by using self-report measures, fits the call for knowledge democratization, as citizens increasingly demand their say in policies and research affecting them (Anderson, 2017; Dedding et al., 2020). This is important, because self-determination can be seen as an essential dimension of quality of life and is linked to many positive outcomes for people with (M)ID) (Schalock et al., 2002; Wehmeyer, 2007; Frielink et al., 2018). Therefore, both the findings of this review and the empirical evidence show that increasing our knowledge of self-report stress measures for people with MID is a highly recommended addition and in line with the contemporary opinion that the voice of people with MID should be included in matters that concern them.

### Measuring the Concept of Stress

The way the concept of stress was operationalized by the self-report measures varied according to the theoretical underpinnings and constructs. Different paradigms or stress theories were used, such as the interactional stress model (e.g., the DSP or the PSS), theories on stress as a transitory anxiety state (e.g., the STAI and the BAI), and the tripartite model of anxiety and depression that describes stress as a common symptom for both (e.g., the DASS). Moreover, some of the self-report measures do not seem to have origins in a certain stress theory or model, but were developed empirically, involving expert consensus on the manifestation of stress in clinical practice (e.g., the GAS-ID). Others are based on the manifestation of stress symptoms described in classification systems of psychiatric disorders (e.g., the SAS-ID). In addition, a distinction can also be seen between self-report stress measures that focus mainly on stress as an experienced psychological and physiological state (e.g., the BAI, the SACL, and the STAI) and those that focus on the experience of stress in the context of situations that actually or hypothetically cause stress, such as job related stress or stressful social situations (e.g., the LI, the PSS, and the PSQ). To ensure that the concepts being studied are consistent with the design and intended use of the self-report measure, we recommend paying attention to how the concept of stress is theoretically framed when deciding to use a self-report stress measure (Cook and Beckman, 2006).

### Implications for Clinical Practice

There is a strong tendency in clinical practice to move away from attributing the symptoms of psychopathology solely to the cognitive deficits of people with MID; this is known as diagnostic overshadowing (Reiss et al., 1982; Hagopian and Jennett, 2008). Clinicians are becoming increasingly sensitive to the fact that people with MID can also suffer from symptoms of psychopathology. Since the degree of stress is now recognized as a significant factor in the development of severe psychopathology, especially in people with MID, it has become more important to correctly assess stress-related states in clinical practice (Scott and Havercamp, 2014). This review therefore provides a practical basis for determining whether and which self-report stress measures are suitable for people with MID within their own clinical context.

To provide some guidance for clinical practice, we have formulated several recommendations based on earlier studies on stress in people with MID and on our own findings from the current review. First, with this review, we want to draw attention to the concept of stress and the importance for clinical practice to consider the degree of (daily or present) stress as a crucial factor in the quality of life and course of further psychological treatment in people with MID. In our view, stress assessment should be included as a regular part of the diagnostic phase of clients with MID when consulting clinical practice. Second, as mentioned, we strongly advise clinical practice to always strive to obtain self-reported information in addition to proxy-reports when it comes to medical, psychological, and service decisions involving people with MID. The third recommendation is based on the results of the current review. We particularly recommend using the three self-report stress measures specifically designed for adults with (M)ID. These self-report measures are characterized by simple Likert rating scales and/or items requiring yes/no responses. Specifically, the use of simpler wording, fewer response options, and the ability to provide supportive visualization are the main differences with the self-report stress measures developed for the non-ID population. Another significant difference is that self-report measures developed for (M)ID often allow the respondent to be assisted during the assessment (SAS-ID) and that the items can be read aloud by someone else (LI and GAS-ID). Although our assessment of the suitability for MID populations show that, even for MID-specific instruments, there is ample room for improvement, these measures remain a clinician's primary choice.

While there is general consensus that it is necessary to timely assess stress in people with MID, we are also aware that this requires experiential knowledge of clinical professionals working with the MID population. The challenge for clinical practice is to prevent that difficult-to-understand behavior of people with MID too quickly leads to a psychiatric classification, which often has far-reaching consequences (Didden et al., 2016). On the other hand, psychological problems still have to be recognized timely. This requires continuous in-depth behavioral observations and careful consideration by clinical professionals, as people with MID, certainly in combination with additional behavioral/psychological problems, often are unable to clearly request help (ten Wolde et al., 2006). Decisions made should therefore be adequately aligned with personal and environmental circumstances, as well as with the level of cognitive functioning (Nouwens et al., 2020). Determining and applying suitable self-report measures for clients with MID could contribute to this purpose. Moreover, as indicated earlier, the use of self-report measures is also a way of letting the client's voice speak, and thereby enhances feelings of autonomy, initiative and freedom of choice. In this study, we have attempted to provide a first guide with regard to the use of self-report stress measures.

### Limitations of the Present Study

There are some limitations of the present study that should be noted. First, because we strictly followed our inclusion criteria, we may have excluded some self-report measures which could be also suitable for assessing stress in people with MID (see Appendix B). For example, they may have not yet been applied in (clinical) outcome studies published in peer-reviewed scientific journals. Searching grey literature sources in addition to the peer-reviewed literature, would probably have resulted in many more measures to include. Another reason for exclusion was that measures were unavailable in the English language; it is possible that suitable measures exist in other languages.

Second, for the appraisal of the psychometric properties of each measure, we had to rely on the parameters reported by authors in their publications. Often, the developers of a measure conducted their own validation research, which may have willingly or unwillingly introduced bias. Nearly all studies report Cronbach's alpha as the main indicator of reliability. Recent advances in psychometric research suggest that this may be a flawed indicator of the internal stability or reliability of a measure. It is stated that other indicators, such as omega, are more robust, and that reliability research should be preceded by Factor Analysis (Crutzen and Peters, 2017).

Third, we would have liked to share more specific information from the expert consultations. However, due to the use of an online survey, there was no opportunity to ask further questions. Therefore, for future research, we recommend adding a more interactive form of data collection when consulting experts on similar questions, such as a multidisciplinary focus group method. Another limitation concerning the expert consultations is that the results reflect the participating experts' professional opinion. Although their clinical and research expertise are highly valued, the experts were not asked to substantiate their statements with references to empirical literature. Therefore, the suggestions by the experts must be valued as tentative and supplementary to the evidence from empirical studies. Finally, the experts' findings were only compared with the published information in the user manuals of the self-report measures, i.e., only with the information already described. An option for follow-up research would be to use a more detailed screening list and to screen the individual instruments with different researchers in the field of MID blinded from each other. This would ensure more accurate statements about the use of existing self-report stress measures in people with MID.

### Implications for Future Research

Our study provides an overview of existing self-report stress measures, but can only offer limited guidance on the suitability of the self-report measures for people with MID. Despite many relevant arguments for the use of self-report measures in intellectual disability research, there are few validated self-report measures available, with even fewer for sensitive topics like stressful experiences (Ruddick and Oliver, 2005; Ali et al., 2008). Information on the suitability of a self-report measure for certain subgroups within the general population such as persons with cognitive impairments, limited verbal abilities, or clinical populations, is generally found in the manual or published peer-reviewed validation research. However, in many cases, self-report measures do not have detailed manuals, the manuals are unavailable, or they do not even exist. We therefore strongly advise future researchers to publish clear user manuals and/or assessment procedures of self-report measures, even if they seem to be simple and easy to use. In addition, for those self-report measures not specifically designed for people with (M)ID, there is no published research on the use in the MID population. Norm data from validity studies are often based on research that excluded people with MID a priori based on their level of IQ. The relevance and suitability of many of the self-report stress measures found for people with MID therefore still remains unclear. More research is needed on the “performance” of a measurement instrument in populations including people with MID. Therefore, we recommend that future validation studies of self-report measures always include a subpopulation composed of respondents with MID.

As noted earlier, stress is operationalized by many different theoretical constructs in the self-report stress measures analyzed. This raises the question of whether this could affect the measured results. On the other hand, research also shows that the operationalization of apparently different concepts, such as “stress” and “state-anxiety,” essentially measure the same items and therefore can be regarded as the same type of outcome (Hook et al., 2008; de Witte et al., 2020a,b). This has led to these concepts being used interchangeably in literature when it comes to outcome studies (Wetsch et al., 2009; Bradt and Dileo, 2014; de Witte et al., 2020b). Nevertheless, we think it essential to provide a theoretical framework underpinning the measurement concepts involved. Not only will this offer the necessary background information for future users, like clinicians, but it also increases the content validity of the self-report measure (Lynn, 1986; Higgins and Straub, 2006).

In order to validly and reliably assess stress-related outcomes in people with MID, attempts should be made to make the self-report stress measures more “MID-inclusive.” However, it is still not entirely clear which specific instrument components or adaptations are required for this purpose. The recent study by Kooijmans et al. (2021a) shows that there are still many gaps to fill on this topic. Findings show, for example, that researchers and clinicians assume questions should be read aloud by the assessor in order to assist people with MID. However, there is reason to believe that this may introduce various forms of bias in the results, arising from complex interviewer-interviewee dynamics (Finlay and Antaki, 2012). More research on the impact of assistance on the outcome of self-report measures is needed to decide whether this is an acceptable practice.

Lastly, the literature shows that Likert scales with three to five answer alternatives can be reliably used in research with people with MID (Fang et al., 2011). However, in the field of stress research, more nuanced response formats may be needed to capture the subtle differences in perceived stress over time. Visual Analogue Scales (VAS), for example, may offer an interesting alternative for this and have potential for assessing stress levels in people with MID. The Subjective Units of Distress Scales (SUDS) developed by Wolpe (1969) is an example of this. Notably, Mevissen et al. (2016) show promising results when using the SUDS in the treatment of trauma-related symptoms of people with MID. As many VAS scales differ in form, more research is advised on how to optimally attune these VAS scale formats to the needs of people with MID.

In conclusion, many adults with MID frequently experience stress in daily life and this has a major impact on their well-being. This emphasizes the importance of assessing stress levels as part of their support needs assessment. Research suggests that self-report measures are more accurate and sensitive compared to proxy measures. However, this scoping review found that there are few self-report stress measures suitable for this purpose.

This underlines the need for continuing efforts to develop high quality and “MID-sensitive” self-report stress measures.

## Author Contributions

All authors listed have made a substantial, direct and intellectual contribution to the work, and approved it for publication.

## Funding

This study was supported by the Nederlandse Organisatie voor Wetenschappelijk Onderzoek (grant number 023.007.068).

## Conflict of Interest

The authors declare that the research was conducted in the absence of any commercial or financial relationships that could be construed as a potential conflict of interest.

## Publisher's Note

All claims expressed in this article are solely those of the authors and do not necessarily represent those of their affiliated organizations, or those of the publisher, the editors and the reviewers. Any product that may be evaluated in this article, or claim that may be made by its manufacturer, is not guaranteed or endorsed by the publisher.

## Appendix A

Exemplary search string for PsycInfo:

(TI (((psycholog^*^ N1 (test OR tests)) OR measur^*^ OR scor^*^) N3 (stress OR “state anxiety”)) OR AB (((psycholog^*^ N1 test^*^) OR measurement) N3 (stress OR “state anxiety”))) NOT post-traumatic.
