# Supplementary material for: Self-Report Stress Measures to Assess Stress in Adults With Mild Intellectual Disabilities—A Scoping Review
Source: Front Psychol. 2021 Oct 25;12:742566. doi: 10.3389/fpsyg.2021.742566 (PMC8573329; doi:10.3389/fpsyg.2021.742566)
Supplement: Supplementary file 1 [file Data_Sheet_1.docx]

Appendix B
*Overview of Stress Self-Report Measures Selected in Step 1*

| **Self-Report Measure** | **Author(s) and publication date** | **Outcome** | **Target group** | **Exclusion reason** |
| --- | --- | --- | --- | --- |
| Beck Anxiety Inventory (BAI) * | Beck et al. (1988). | State anxiety | Adults and people with ID |  |
| Cognitive Anxiety Scale-State | Weinstein & Smith (1987) | Stress | No information available | No published studies or psychometric data available |
| Computerized Stress Inventory | Press & Osterkamp (1984) | Stress  (stress levels and life satisfaction) | Adults | No published studies or psychometric data available |
| Depression Anxiety Stress Scales (DASS) * | Lovibond & Lovibond (1995). | Stress / state anxiety | Adults |  |
| Derogatis Stress Profile (DSP) | Derogatis (1980) | Stress | Adults |  |
| Glasgow Anxiety Scale* (GAS-ID) | Mindham & Espie (2003) | State anxiety | “People with an intellectual disability” (age not specified) |  |
| Hamilton Anxiety Rating Scale (HAM-A) | Hamilton (1959) | State anxiety | Adults with diagnosis of anxiety disorder | Developed for adults with a specific diagnosis |
| Index of Clinical Stress (ICS) * | Abell (1991) | Stress | Adults & youths age 12+ |  |
| Lifestress Inventory (LI) * | Bramston & Fogarty (1997) | Stress | Suitable for a wide range of people, including MID |  |
| Pikunas Adult Stress Inventory (PASI) | Pikunas (1984) | Stress | Adults & youths age 16+ | No published studies or psychometric data available |
| Psychological Stress Measure (PSM-9 ) * | Tessier et al. (1990) | Stress | Adults |  |
| Perceived Stress Questionnaire (PSQ) * | Levenstein et al. (1993) | Stress | Adult |  |
| Perceived Stress Reactivity Scale (PSRS) | Schlotz et al. (2011) | Stress | Adults | Anticipatory & specific situations |
| Perceived Stress Scale (PSS) * | Cohen et al. (1983) | Stress | Adults |  |
| Recovery-Stress-Questionnaires (RESTQ) | Kallus & Kellmann (2016) | Stress | Adults, separate version for adolescents and children | Specific situations: stress during recreational activities |
| Stress Arousal Checklist (SACL) * | Mackay et al. (1978) | Stress and arousal | Adults |  |
| Self-Rating Anxiety Scale for  adults with Intellectual Disabilities (SAS-ID) * | Zung (1971)  ID version: Lindsay & Michie (1988) | State anxiety | Adults with ID |  |
| Stress Evaluation Inventory (SEI) | Kulhavy & Dee-Burnett (1984). | Stress (career, family, and personal-social) | Adults | No published studies or psychometric data available |
| Stress Overload Scale (SOS) * | Amirkhan (2012) | Stress | Adults |  |
| Stress Response Inventory (SRI) | Koh et al. (2001) | Stress | Adults | No information on English version available |
| Stress Symptom Checklist (SSCL) * | Schlebusch (2004) | Stress levels | Adults |  |
| Subjective Stress Scale (SSS) | Bramston & Bostock (1994) | Stress | Adults with ID | Updated version of SSS is included (LI) |
| State version of the State-Trait Anxiety  Inventory (STAI-S) * | Spielberger (1981) | State anxiety | Adults |  |
| Stress Analysis System (SAS) | Nelson et al. (1983) | Stress | No information found | No published studies or psychometric data available |
| Subjective Units of Distress Scale (SUDS) | Wolpe (1969) | Stress | Adults, adolescents and children, including persons with ID | Not a standardized measure, but a flexible clinical and research outcome tool |

* included in final analysis
